# Supplementary material for: Pyruvate Dehydrogenase Kinase 1 inhibition mediated oxidative phosphorylation enhancement in cartilage promotes osteoarthritis progression
Source: BMC Musculoskelet Disord. 2023 Jul 20;24:597. doi: 10.1186/s12891-023-06585-6 (PMC10357736; doi:10.1186/s12891-023-06585-6)
Supplement: Supplementary file 1 — Additional file 1. [file 12891_2023_6585_MOESM1_ESM.zip › MF.Gsea.1653627316242/GOMF_CATALYTIC_ACTIVITY_ACTING_ON_A_TRNA.html]

Details for gene set GOMF\_CATALYTIC\_ACTIVITY\_ACTING\_ON\_A\_TRNA[GSEA]

|  || Dataset | 55457\_collapsed\_to\_symbols.cls.cls #OA\_versus\_normal.cls.cls #OA\_versus\_normal\_repos |
| Phenotype | cls.cls#OA\_versus\_normal\_repos |
| Upregulated in class | OA |
| GeneSet | GOMF\_CATALYTIC\_ACTIVITY\_ACTING\_ON\_A\_TRNA |
| Enrichment Score (ES) | 0.28308177 |
| Normalized Enrichment Score (NES) | 1.2921578 |
| Nominal p-value | 0.05820106 |
| FDR q-value | 0.41022635 |
| FWER p-Value | 1.0 |
Table: GSEA Results Summary

  

Fig 1: Enrichment plot: GOMF\_CATALYTIC\_ACTIVITY\_ACTING\_ON\_A\_TRNA      
 Profile of the Running ES Score & Positions of GeneSet Members on the Rank Ordered List

  

| SYMBOL | TITLE | RANK IN GENE LIST | RANK METRIC SCORE | RUNNING ES | CORE ENRICHMENT || 1 | DALRD3 | DALR anticodon binding domain containing 3 [Source:HGNC Symbol;Acc:HGNC:25536] | 71 | 0.957 | 0.0284 | Yes |
| 2 | TRMT5 | tRNA methyltransferase 5 [Source:HGNC Symbol;Acc:HGNC:23141] | 125 | 0.879 | 0.0554 | Yes |
| 3 | RPP30 | ribonuclease P/MRP subunit p30 [Source:HGNC Symbol;Acc:HGNC:17688] | 265 | 0.777 | 0.0721 | Yes |
| 4 | POP5 | "POP5 homolog, ribonuclease P/MRP subunit [Source:HGNC Symbol;Acc:HGNC:17689]" | 494 | 0.662 | 0.0777 | Yes |
| 5 | SARS1 | seryl-tRNA synthetase 1 [Source:HGNC Symbol;Acc:HGNC:10537] | 516 | 0.654 | 0.0993 | Yes |
| 6 | TYW1 | tRNA-yW synthesizing protein 1 homolog [Source:HGNC Symbol;Acc:HGNC:25598] | 523 | 0.650 | 0.1219 | Yes |
| 7 | EPRS1 | glutamyl-prolyl-tRNA synthetase 1 [Source:HGNC Symbol;Acc:HGNC:3418] | 581 | 0.629 | 0.1397 | Yes |
| 8 | RPP14 | ribonuclease P/MRP subunit p14 [Source:HGNC Symbol;Acc:HGNC:30327] | 835 | 0.559 | 0.1398 | Yes |
| 9 | DUS4L | dihydrouridine synthase 4 like [Source:HGNC Symbol;Acc:HGNC:21517] | 862 | 0.552 | 0.1573 | Yes |
| 10 | LCMT2 | leucine carboxyl methyltransferase 2 [Source:HGNC Symbol;Acc:HGNC:17558] | 946 | 0.529 | 0.1696 | Yes |
| 11 | PRORP | protein only RNase P catalytic subunit [Source:HGNC Symbol;Acc:HGNC:19958] | 1013 | 0.515 | 0.1827 | Yes |
| 12 | POP4 | "POP4 homolog, ribonuclease P/MRP subunit [Source:HGNC Symbol;Acc:HGNC:30081]" | 1015 | 0.514 | 0.2008 | Yes |
| 13 | HARS2 | "histidyl-tRNA synthetase 2, mitochondrial [Source:HGNC Symbol;Acc:HGNC:4817]" | 1195 | 0.478 | 0.2038 | Yes |
| 14 | QRSL1 | glutaminyl-tRNA amidotransferase subunit QRSL1 [Source:HGNC Symbol;Acc:HGNC:21020] | 1250 | 0.467 | 0.2161 | Yes |
| 15 | MRPL58 | mitochondrial ribosomal protein L58 [Source:HGNC Symbol;Acc:HGNC:5359] | 1298 | 0.459 | 0.2287 | Yes |
| 16 | FTO | FTO alpha-ketoglutarate dependent dioxygenase [Source:HGNC Symbol;Acc:HGNC:24678] | 1314 | 0.456 | 0.2437 | Yes |
| 17 | POP7 | "POP7 homolog, ribonuclease P/MRP subunit [Source:HGNC Symbol;Acc:HGNC:19949]" | 1337 | 0.452 | 0.2580 | Yes |
| 18 | DARS1 | aspartyl-tRNA synthetase 1 [Source:HGNC Symbol;Acc:HGNC:2678] | 1558 | 0.419 | 0.2557 | Yes |
| 19 | IARS2 | "isoleucyl-tRNA synthetase 2, mitochondrial [Source:HGNC Symbol;Acc:HGNC:29685]" | 1685 | 0.401 | 0.2600 | Yes |
| 20 | FARS2 | "phenylalanyl-tRNA synthetase 2, mitochondrial [Source:HGNC Symbol;Acc:HGNC:21062]" | 1686 | 0.401 | 0.2742 | Yes |
| 21 | TRMT11 | tRNA methyltransferase 11 homolog [Source:HGNC Symbol;Acc:HGNC:21080] | 1885 | 0.374 | 0.2721 | Yes |
| 22 | QARS1 | glutaminyl-tRNA synthetase 1 [Source:HGNC Symbol;Acc:HGNC:9751] | 1913 | 0.371 | 0.2831 | Yes |
| 23 | GATB | glutamyl-tRNA amidotransferase subunit B [Source:HGNC Symbol;Acc:HGNC:8849] | 2515 | 0.302 | 0.2469 | No |
| 24 | NARS2 | "asparaginyl-tRNA synthetase 2, mitochondrial [Source:HGNC Symbol;Acc:HGNC:26274]" | 2631 | 0.287 | 0.2481 | No |
| 25 | FTSJ1 | FtsJ RNA 2'-O-methyltransferase 1 [Source:HGNC Symbol;Acc:HGNC:13254] | 2664 | 0.284 | 0.2556 | No |
| 26 | WARS2 | "tryptophanyl tRNA synthetase 2, mitochondrial [Source:HGNC Symbol;Acc:HGNC:12730]" | 3014 | 0.248 | 0.2372 | No |
| 27 | ELAC1 | elaC ribonuclease Z 1 [Source:HGNC Symbol;Acc:HGNC:14197] | 3108 | 0.239 | 0.2384 | No |
| 28 | DUS2 | dihydrouridine synthase 2 [Source:HGNC Symbol;Acc:HGNC:26014] | 3182 | 0.233 | 0.2409 | No |
| 29 | TRMT12 | tRNA methyltransferase 12 homolog [Source:HGNC Symbol;Acc:HGNC:26091] | 3208 | 0.230 | 0.2471 | No |
| 30 | SARS2 | "seryl-tRNA synthetase 2, mitochondrial [Source:HGNC Symbol;Acc:HGNC:17697]" | 3411 | 0.210 | 0.2388 | No |
| 31 | NSUN3 | NOP2/Sun RNA methyltransferase 3 [Source:HGNC Symbol;Acc:HGNC:26208] | 3612 | 0.192 | 0.2300 | No |
| 32 | TSEN34 | tRNA splicing endonuclease subunit 34 [Source:HGNC Symbol;Acc:HGNC:15506] | 3617 | 0.192 | 0.2365 | No |
| 33 | TRMT1L | tRNA methyltransferase 1 like [Source:HGNC Symbol;Acc:HGNC:16782] | 4007 | 0.155 | 0.2116 | No |
| 34 | RPP21 | ribonuclease P/MRP subunit p21 [Source:HGNC Symbol;Acc:HGNC:21300] | 4269 | 0.131 | 0.1959 | No |
| 35 | DARS2 | "aspartyl-tRNA synthetase 2, mitochondrial [Source:HGNC Symbol;Acc:HGNC:25538]" | 4480 | 0.115 | 0.1836 | No |
| 36 | ELAC2 | elaC ribonuclease Z 2 [Source:HGNC Symbol;Acc:HGNC:14198] | 4532 | 0.110 | 0.1835 | No |
| 37 | NARS1 | asparaginyl-tRNA synthetase 1 [Source:HGNC Symbol;Acc:HGNC:7643] | 4651 | 0.100 | 0.1778 | No |
| 38 | ALKBH1 | "alkB homolog 1, histone H2A dioxygenase [Source:HGNC Symbol;Acc:HGNC:17911]" | 4765 | 0.090 | 0.1722 | No |
| 39 | HARS1 | histidyl-tRNA synthetase 1 [Source:HGNC Symbol;Acc:HGNC:4816] | 5196 | 0.057 | 0.1406 | No |
| 40 | RARS1 | arginyl-tRNA synthetase 1 [Source:HGNC Symbol;Acc:HGNC:9870] | 5469 | 0.035 | 0.1207 | No |
| 41 | TARS2 | "threonyl-tRNA synthetase 2, mitochondrial [Source:HGNC Symbol;Acc:HGNC:30740]" | 5576 | 0.026 | 0.1133 | No |
| 42 | TRMT9B | tRNA methyltransferase 9B (putative) [Source:HGNC Symbol;Acc:HGNC:26725] | 5702 | 0.016 | 0.1041 | No |
| 43 | AARS1 | alanyl-tRNA synthetase 1 [Source:HGNC Symbol;Acc:HGNC:20] | 5703 | 0.015 | 0.1047 | No |
| 44 | METTL8 | "methyltransferase 8, methylcytidine [Source:HGNC Symbol;Acc:HGNC:25856]" | 5706 | 0.015 | 0.1050 | No |
| 45 | THUMPD2 | THUMP domain containing 2 [Source:HGNC Symbol;Acc:HGNC:14890] | 5913 | 0.001 | 0.0890 | No |
| 46 | TRDMT1 | tRNA aspartic acid methyltransferase 1 [Source:HGNC Symbol;Acc:HGNC:2977] | 6204 | -0.021 | 0.0671 | No |
| 47 | TRMT61B | tRNA methyltransferase 61B [Source:HGNC Symbol;Acc:HGNC:26070] | 6223 | -0.022 | 0.0665 | No |
| 48 | POP1 | "POP1 homolog, ribonuclease P/MRP subunit [Source:HGNC Symbol;Acc:HGNC:30129]" | 6261 | -0.025 | 0.0645 | No |
| 49 | TSEN2 | tRNA splicing endonuclease subunit 2 [Source:HGNC Symbol;Acc:HGNC:28422] | 6331 | -0.031 | 0.0602 | No |
| 50 | MARS1 | methionyl-tRNA synthetase 1 [Source:HGNC Symbol;Acc:HGNC:6898] | 6400 | -0.035 | 0.0561 | No |
| 51 | METTL2B | "methyltransferase 2B, methylcytidine [Source:HGNC Symbol;Acc:HGNC:18272]" | 6522 | -0.044 | 0.0483 | No |
| 52 | RPP38 | ribonuclease P/MRP subunit p38 [Source:HGNC Symbol;Acc:HGNC:30329] | 6814 | -0.064 | 0.0278 | No |
| 53 | KARS1 | lysyl-tRNA synthetase 1 [Source:HGNC Symbol;Acc:HGNC:6215] | 6837 | -0.066 | 0.0284 | No |
| 54 | TRMT2B | tRNA methyltransferase 2 homolog B [Source:HGNC Symbol;Acc:HGNC:25748] | 6855 | -0.068 | 0.0295 | No |
| 55 | LARS2 | "leucyl-tRNA synthetase 2, mitochondrial [Source:HGNC Symbol;Acc:HGNC:17095]" | 7017 | -0.081 | 0.0198 | No |
| 56 | GARS1 | glycyl-tRNA synthetase 1 [Source:HGNC Symbol;Acc:HGNC:4162] | 7069 | -0.085 | 0.0189 | No |
| 57 | CDKAL1 | CDK5 regulatory subunit associated protein 1 like 1 [Source:HGNC Symbol;Acc:HGNC:21050] | 7761 | -0.138 | -0.0302 | No |
| 58 | GATC | glutamyl-tRNA amidotransferase subunit C [Source:HGNC Symbol;Acc:HGNC:25068] | 7852 | -0.144 | -0.0321 | No |
| 59 | TARBP1 | TAR (HIV-1) RNA binding protein 1 [Source:HGNC Symbol;Acc:HGNC:11568] | 8091 | -0.163 | -0.0449 | No |
| 60 | RPP40 | ribonuclease P/MRP subunit p40 [Source:HGNC Symbol;Acc:HGNC:20992] | 8318 | -0.179 | -0.0562 | No |
| 61 | TRMO | tRNA methyltransferase O [Source:HGNC Symbol;Acc:HGNC:30967] | 8541 | -0.197 | -0.0665 | No |
| 62 | QTRT1 | queuine tRNA-ribosyltransferase catalytic subunit 1 [Source:HGNC Symbol;Acc:HGNC:23797] | 8620 | -0.202 | -0.0654 | No |
| 63 | TRMU | tRNA mitochondrial 2-thiouridylase [Source:HGNC Symbol;Acc:HGNC:25481] | 8916 | -0.226 | -0.0805 | No |
| 64 | CARS2 | "cysteinyl-tRNA synthetase 2, mitochondrial [Source:HGNC Symbol;Acc:HGNC:25695]" | 8958 | -0.228 | -0.0756 | No |
| 65 | CARS1 | cysteinyl-tRNA synthetase 1 [Source:HGNC Symbol;Acc:HGNC:1493] | 9062 | -0.237 | -0.0752 | No |
| 66 | YARS1 | tyrosyl-tRNA synthetase 1 [Source:HGNC Symbol;Acc:HGNC:12840] | 9422 | -0.266 | -0.0938 | No |
| 67 | WDR4 | WD repeat domain 4 [Source:HGNC Symbol;Acc:HGNC:12756] | 9631 | -0.286 | -0.0999 | No |
| 68 | PTRH2 | peptidyl-tRNA hydrolase 2 [Source:HGNC Symbol;Acc:HGNC:24265] | 9707 | -0.293 | -0.0954 | No |
| 69 | LRRC47 | leucine rich repeat containing 47 [Source:HGNC Symbol;Acc:HGNC:29207] | 10062 | -0.327 | -0.1114 | No |
| 70 | IARS1 | isoleucyl-tRNA synthetase 1 [Source:HGNC Symbol;Acc:HGNC:5330] | 10154 | -0.337 | -0.1066 | No |
| 71 | AARSD1 | alanyl-tRNA synthetase domain containing 1 [Source:HGNC Symbol;Acc:HGNC:28417] | 10222 | -0.343 | -0.0997 | No |
| 72 | WARS1 | tryptophanyl-tRNA synthetase 1 [Source:HGNC Symbol;Acc:HGNC:12729] | 10289 | -0.350 | -0.0924 | No |
| 73 | YARS2 | tyrosyl-tRNA synthetase 2 [Source:HGNC Symbol;Acc:HGNC:24249] | 10299 | -0.350 | -0.0807 | No |
| 74 | METTL1 | "methyltransferase 1, tRNA methylguanosine [Source:HGNC Symbol;Acc:HGNC:7030]" | 10333 | -0.354 | -0.0707 | No |
| 75 | DTWD1 | DTW domain containing 1 [Source:HGNC Symbol;Acc:HGNC:30926] | 10549 | -0.378 | -0.0741 | No |
| 76 | TRMT61A | tRNA methyltransferase 61A [Source:HGNC Symbol;Acc:HGNC:23790] | 10603 | -0.384 | -0.0647 | No |
| 77 | RPP25 | ribonuclease P and MRP subunit p25 [Source:HGNC Symbol;Acc:HGNC:30361] | 10621 | -0.385 | -0.0523 | No |
| 78 | VARS1 | valyl-tRNA synthetase 1 [Source:HGNC Symbol;Acc:HGNC:12651] | 11035 | -0.435 | -0.0692 | No |
| 79 | TARS1 | threonyl-tRNA synthetase 1 [Source:HGNC Symbol;Acc:HGNC:11572] | 11357 | -0.480 | -0.0772 | No |
| 80 | QTRT2 | queuine tRNA-ribosyltransferase accessory subunit 2 [Source:HGNC Symbol;Acc:HGNC:25771] | 11420 | -0.489 | -0.0647 | No |
| 81 | PUS3 | pseudouridine synthase 3 [Source:HGNC Symbol;Acc:HGNC:25461] | 11503 | -0.501 | -0.0533 | No |
| 82 | TRIT1 | tRNA isopentenyltransferase 1 [Source:HGNC Symbol;Acc:HGNC:20286] | 11658 | -0.526 | -0.0467 | No |
| 83 | TRMT1 | tRNA methyltransferase 1 [Source:HGNC Symbol;Acc:HGNC:25980] | 11775 | -0.545 | -0.0364 | No |
| 84 | TRMT13 | tRNA methyltransferase 13 homolog [Source:HGNC Symbol;Acc:HGNC:25502] | 11792 | -0.548 | -0.0183 | No |
| 85 | PUS1 | pseudouridine synthase 1 [Source:HGNC Symbol;Acc:HGNC:15508] | 12260 | -0.664 | -0.0312 | No |
| 86 | DUS1L | dihydrouridine synthase 1 like [Source:HGNC Symbol;Acc:HGNC:30086] | 12355 | -0.693 | -0.0140 | No |
| 87 | LARS1 | leucyl-tRNA synthetase 1 [Source:HGNC Symbol;Acc:HGNC:6512] | 12415 | -0.712 | 0.0066 | No |
| 88 | FARSA | phenylalanyl-tRNA synthetase subunit alpha [Source:HGNC Symbol;Acc:HGNC:3592] | 12714 | -0.878 | 0.0145 | No |
Table: GSEA details [plain text format]

  

Fig 2: GOMF\_CATALYTIC\_ACTIVITY\_ACTING\_ON\_A\_TRNA      
 Blue-Pink O' Gram in the Space of the Analyzed GeneSet

  

Fig 3: GOMF\_CATALYTIC\_ACTIVITY\_ACTING\_ON\_A\_TRNA: Random ES distribution      
 Gene set null distribution of ES for **GOMF\_CATALYTIC\_ACTIVITY\_ACTING\_ON\_A\_TRNA**
